# Cathepsin C Is Involved in Macrophage M1 Polarization via p38/MAPK Pathway in Sudden Cardiac Death

**DOI:** 10.1155/2021/6139732

**Published:** 2021-10-15

**Authors:** Jialin Dai, Jiangjin Liu, Qiong Zhang, Yang An, Bing Xia, Changwu Wan, Yuanyuan Zhang, Yanni Yu, Jie Wang

**Affiliations:** School of Forensic Medicine, Guizhou Medical University, 4 Beijing Road, Guiyang, 550001 Guizhou, China

## Abstract

This study was aimed at identifying molecular markers associated with the pathogenesis of sudden cardiac death (SCD). It provides a proteomic analysis of human left anterior descending coronary artery from subjects diagnosed with SCD through histological examination and cases of nondisease accidental deaths through autopsy. A total of 2784 proteins were obtained from label-free quantitative proteomic analysis. This included a total of 265 differential proteins which were involved in SCD-related processes, such as inflammation, muscle system process regulation, metal ion transport, and lysosomal pathway. Western blotting was carried out to measure the expressions of cathepsin C (CTSC), focal adhesion kinase (FAK), p-FAK, and proteins related to the p38/MAPK signaling pathway, whereas immunohistochemistry was performed to determine the localization and expression of CTSC, TNF-*α*, and CD206 in arterial tissues. It was found that CTSC were the most expressed proteins with a significant upward trend in SCD cases. Besides, CTSC regulated macrophage polarization to M1 through the FAK-induced p38/MAPK signaling pathway. This promoted the release of inflammatory factors and eventually increased the inflammatory response. In conclusion, this study implies that CTSC may be one of the key molecular targets for promoting macrophage M1 polarization in SCD, which may provide new therapeutic insights into the treatment of inflammatory diseases.

## 1. Introduction

Sudden cardiac death (SCD) is an accidental death caused by a sudden cessation of cardiac activity. According to the World Health Organization (WHO), SCD is defined as a sudden death within 1 hour of the onset or within 24 hours of last being seen alive. Among SCD cases of patients over the age of 35, up to 30% of them cannot be explained even with forensic analysis. There are multiple factors that lead to the development of sudden cardiac death. Coronary atherosclerosis is regarded as the most common pathological basis of SCD. It can cause myocardial infarction and different types of arrhythmias leading to sudden death, as a result of the reduction or interruption of coronary blood flow. The diagnosis, treatment, and prevention of SCD have been improved due to a long-term understanding of the relationship between atherosclerosis and SCD in anatomic pathology and pathophysiology. However, the incidence remains relatively consistent in the overall cardiovascular death [1], and the pathogenesis should be fully clarified [2, 3]. In this context, it is imperative to identify and further understand potential molecular markers for improvements in SCD prevention.

Several research studies have identified the important role of proteome in interpreting the occurrence and development of diseases. This is because proteome can describe proteins in different physiological or pathological states. Herrington et al. [4] studied proteomic features in 100 adults after autopsy and identified protein biomarkers of early atherosclerosis. Elsewhere, Doll et al. [5] conducted a proteomic study in 3 healthy heart samples with 16 different anatomical regions and 4 types of heart cells. They mapped the results to find proteomic differences in a region-dependent manner and suggested variance in protein functions. This study applied label-free quantitative proteomics in observing the changes of protein abundance in the left anterior descending coronary artery of each selected case. The method can achieve relatively deep analysis and wide dynamic range, and results are valuable for understanding the pathogenesis of SCD. Additionally, high-performance liquid chromatography-mass spectrometry (HPLC-MS) analysis identified a total of 29,581 peptide fragments and 2,784 quantifiable proteins. Six proteins were selected as the core proteins which are potentially related to the occurrence of SCD. Among these proteins, CTSC was significantly increased in SCD. This was validated in the subsequent phase. Therefore, CTSC was identified to promote inflammatory response through inducing macrophage polarization to M1. This indicates that CTSC may play an important role in the progress of SCD.

## 2. Materials and Methods

### 2.1. Experimental Design

Sudden cardiac death (SCD) subjects (*n* = 3) and non-SCD (*n* = 3) were selected to perform quantitative proteomic analysis of human left anterior descending (LAD) coronary artery associated with SCD in this study. The inclusion criteria of SCD were that autopsy subjects were diagnosed with “coronary heart disease” by histological examination. The anterior descending branch of the left coronary artery has atheromatous plaque formation and lumen stenosis ≥ 50% and death within 24 hours, excluding violent death and other fatal diseases. Explicit exclusion criteria were (1) the time of death being more than 24 hours (from death to autopsy); (2) the deceased suffered from other types of heart disease, including dilated cardiomyopathy, Kashan disease, myocarditis, hypothyroid heart disease, constrictive pericarditis, and amyloidosis of the heart; and (3) drug abuse.

**Figure 1:**
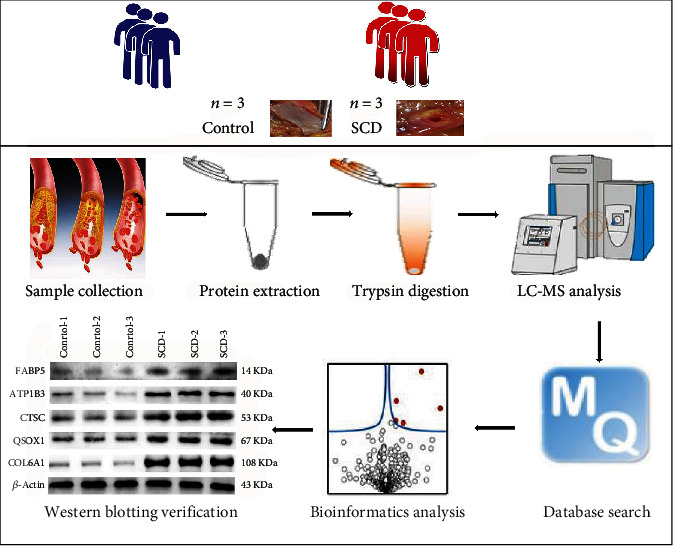
Experimental workflow of quantitative proteomic analysis of human left anterior descending coronary artery (LAD) using label-free quantitative proteomic approach.

The inclusion criteria of the control were the causes of death being accidental such as falling, traffic accidents, and electric shocks, pathology examination not revealing any heart disease or coronary stenosis, and death also within 24 hours. The exclusion criteria for the control group were the same as previously described for the SCD group. The study design was as illustrated in Figure 1. Label-free quantitative proteomic analyses were used to analyze the samples. After, protein extraction and trypsin digestion were done and then analyzed through timsTOF Pro mass spectrometry. The Maxquant search engine (v.1.6.6.0) was utilized in the processing of MS/MS data. In addition to proteomic analysis using bioinformatics, the expression levels of specific DEPs by Western blot and functional verification were also performed.

### 2.2. Sampling of Human Coronary Arteries

This study used samples of coronary arteries obtained from autopsy samples. The samples were collected between October 2018 and December 2019 from the Forensic Judicial Expertise Center of Guizhou Medical University. The samples were used to confirm the validity of the DEPs through label-free quantitative proteomic analysis. A total of 79 samples were included in the study based on the inclusion and exclusion criteria. The samples were divided into three groups based on CHD diagnosis and the causes of the death: control group (*n* = 25), CHD+non-SCD group (*n* = 26), and CHD+SCD group (*n* = 28).

Statements of informed consent could not be obtained from most of the patients because they had died or had been lost to follow-up. However, ethical approval of this study was obtained from the Ethics Committee of Guizhou Medical University (Approval Number: 2021-62).

### 2.3. Protein Extraction and Trypsin Digestion of Coronary Arteries

For protein isolation, frozen samples were first ground into cell powder using liquid nitrogen and then transferred to a centrifuge tube (5 mL). Subsequently, four volumes of lysis buffer (1% protease inhibitor, 8 M urea) were added to the grounded cell powder before sonicating the solution thrice in ice using a high-intensity ultrasonic processor. To get rid of the debris, the solution was centrifuged (12,000 rpm at 4°C) for 10 minutes and the supernatant was subsequently collected. Finally, the BCA kit was used to determine the concentration of the protein according to methods described by the manufacturer.

For digestion, 5 mM dithiothreitol was used to reduce the protein solution for 30 minutes at 56°C. The solution was then alkylated with 11 mM iodoacetamide in darkness and at room temperature for 15 minutes. Protein samples were diluted by adding 100 mM TEAB to urea (<2 M concentration). In the final step, the protein samples were digested overnight by trypsin at a ratio of 1 : 50 (trypsin-to-protein mass ratio) and then for 4 hours at a ratio of 1 : 100 (trypsin-to-protein mass ratio).

### 2.4. LC-MS/MS Analysis and Database Search

The peptides were dissolved with liquid chromatography mobile phase A (0.1% (*v*/*v*) formic acid aqueous solution) and then separated through the NanoElute ultra-high-performance liquid phase system. The two LC buffers used were buffer A (0.1% (*v*/*v*) formic acid in Milli-Q water) and buffer B (0.1% formic acid in 90% acetonitrile).

The peptides were separated by an ultraefficient liquid phase system. They were injected into a capillary ion source for ionization and then into timsTOF Pro mass spectrometry for analysis. After a first-stage mass spectrometry acquisition, secondary spectrogram with the charge of the parent ion in the range of 0 to 5 was collected in the PASEF mode for 10 times.

### 2.5. Bioinformatics Methods

The resulting MS/MS data were processed using the Maxquant search engine (v.1.6.6.0). For the search parameter settings: the database used was Human_SwissPro, and an inverse library was added to calculate the false positive rate (FDR) due to random matching. To eliminate the effect of contaminated proteins in identification results, a common contamination library was then added to the database. Trypsin/P was specified as a cleavage enzyme allowing up to 2 missing cleavages. Cysteine alkylation was specified as fixed modification and oxidation of methionine whereas acetylation of n-terminal protein and deamidation (NQ) were specified as variable modifications. FDR was adjusted to <1%.

The principal component analysis (PCA) was used to assess the quantitative reproducibility of proteins. Differentially expressed proteins (DEPs) were identified using Limma from R (version: 4.0), and the values for statistical significance were set as *p* value < 0.05 and 1.5-fold changes. Volcano maps and functional enrichment were performed using Hiplot (https://hiplot.com.cn). The protein-protein interaction (PPI) network was constructed through the STRING (https://string-db.org/) (version 11.0). The plug-in Molecular Complex Detection (MCODE) was then employed to screen the modules of the PPI network in Cytoscape [6] (version 3.4.0, http://chianti.ucsd.edu/cytoscape-3.4.0/).

### 2.6. Western Blot Validation

The protein of coronary artery tissues was separated by 12% sodium dodecyl sulfate polyacrylamide gel electrophoresis (SDS-PAGE) and transferred onto the polyvinylidene fluoride (PVDF) membrane by electroblotting. The membrane was subsequently incubated with a monoclonal antibody. They were then subjected to horseradish peroxidase- (HRP-) labeled goat anti-mouse IgG polymer (1 : 8000, Solarbio, China) and horseradish peroxidase- (HRP-) labeled goat anti-rabbit IgG polymer (1 : 5000, Thermo Fisher, USA). The purpose and *β*-actin grayscale values were measured after the addition of developer and postexposure. The values were then analyzed using ImageJ analysis software (National Institutes of Health, USA).

### 2.7. Immunohistochemistry

Immunohistochemical (IHC) studies were conducted through the streptavidin-peroxidase complex method. Paraffin sections of coronary arteries were incubated overnight at 4°C with primary antibodies (CTSC (1 : 200, Abcam, USA) and TNF-*α* (1 : 100, Abcam, USA)). The sections were then incubated with biotin-labelled secondary antibodies. Lastly, diaminobenzidine (DAB) was used for color reaction. Images were taken using Image-Pro Plus 6.0 software.

### 2.8. Statistical Analysis

The data from this study were analyzed using SPSS 22.0 software. Continuous variables were presented as the mean ± standard deviation (SD), whereas categorical variables were presented as frequencies and percentages. One-way ANOVA was performed for comparison of multiple groups whereas *T*-tests were used to compare data for continuous variables between two groups. The optical density of CTSC measured by immunohistochemistry was used as an independent variable and CHD and SCD as the outcome variable to plot the ROC (receiver operating characteristic) and record the AUC (area under the ROC curve). All two-tailed *p* < 0.05 was taken as statistically significant.

## 3. Results

### 3.1. Left Anterior Descending Coronary Artery (LAD) Proteome Profile Associated with SCD

A total of 274723.0 spectrograms were obtained. After searching the Human_SwissProt database, 151892 spectra were matched to 29581.0 peptides in the database. Among the 151892 matched spectra, the specific peptide was 26720. A total of 3678 proteins were identified. It was found that the quantifiable proteins were 2784. The quantitative proteins were indicating the quantitative information in at least one comparison group. Within the sample repeatability test, we consider the intragroup data to be acceptable by PCA (Supplementary Figure 1).

**Figure 2:**
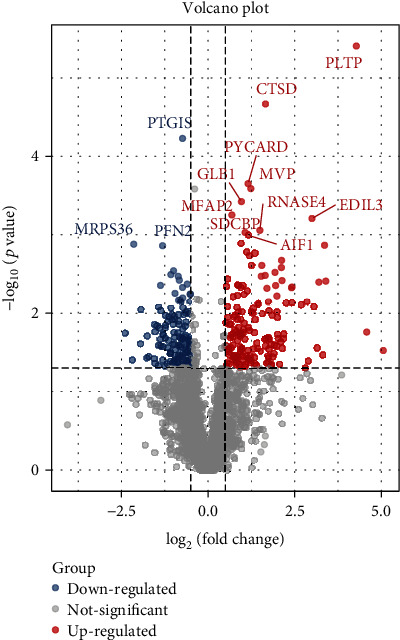
Differential gene expression analysis. The volcano plots showed the differentially expressed proteins in the left anterior descending coronary branch tissues of SCD patients compared with normal.

After Limma analysis in the R package, 265 differentially expressed proteins were found according to the 1.5-fold changes (*p* < 0.05). This also included 156 upregulated DEPs and 109 downregulated DEPs. Therefore, the results of the DEGs were used for subsequent analysis. The volcano plot shown in Figure 2 demonstrates the distributions of statistical significance and magnitude of change for these proteins in the different groups.

### 3.2. Annotation Analysis of the Differentially Expressed Proteins Associated with SCD

**Figure 3:**
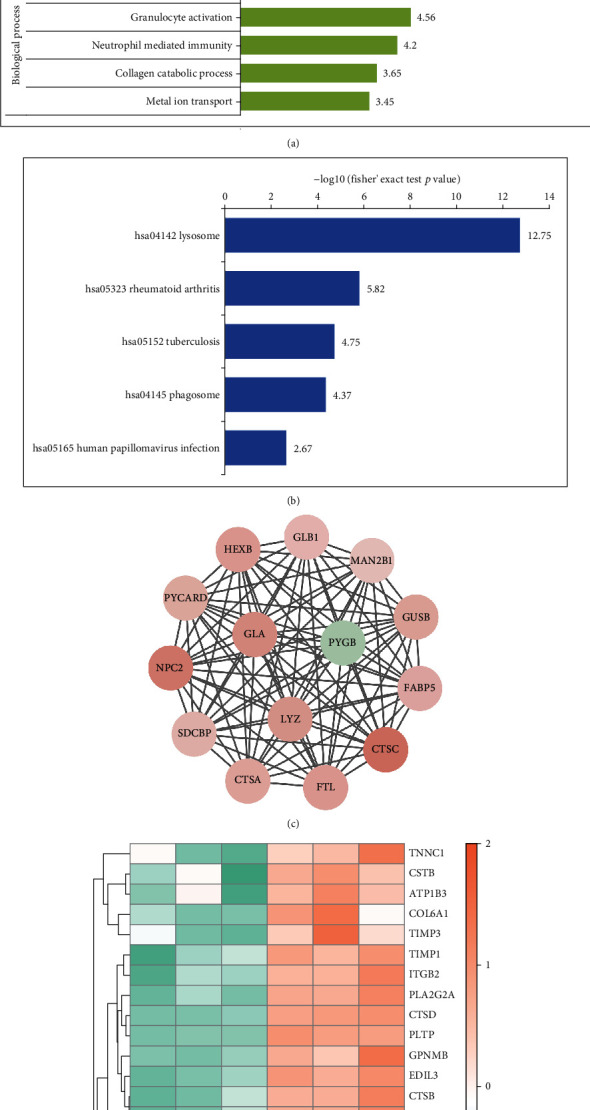
Function enrichment and the protein-protein interaction (PPI) network module, analysis, and hub gene selection. (a, b) Detailed information relating to changes in the biological processes (BP), cellular components (CC), molecular functions (MF), and the KEGG pathway analysis of DEGs. (c) Results of subnet module I analysis of the PPI network. (d) Some hub protein heat map performed with hierarchical clustering. Orange represents upregulated genes, and green represents downregulated genes.

Differentially expressed genes (DEGs) were analyzed to further the insight of the functions and characteristics of identified DEGs in SCD. The proteins were classified by GO and KEGG. Further, in comparison with the control group, the key potentially critical biological processes and pathways that might discriminate against SCD were identified. The results of biological process (BP) showed that the DEPs were invalid in immune response, reactive oxygen metabolism, and lipid metabolism. It was also found that, according to cellular compartment (CC) annotation, the majority of the dysregulated proteins originated from extracellular space, lysosomal lumen, vacuolar lumen, and intracellular vesicle. Moreover, analysis of the KEGG pathway showed that the DEPs were primarily enriched in lysosome, rheumatoid arthritis, tuberculosis, vascular smooth muscle contraction, and so on (Figures 3(a) and 3(b)).

### 3.3. Network Analysis Identifies SCD-Related Core Proteins

String software was used to construct the PPI network for a better understanding of the relationships between protein-protein relationships. According to the principle of the minimum required interaction score with medium confidence of >0.7, the PPI network of DEPs consisted of 162 nodes and 497 edges. This included 112 upregulated proteins and 50 downregulated proteins. The PPI was visualized in Cytoscape, and the core modules were filtered using the MCODE plugin. A total of 5 significant modules with 127 proteins were found after selection of the combined score ≥ 5 in the plugin (Figure 3(c)). These modules were concentrated in lysosomes and phagosomes with involvement in pathways related to inflammation, antigen processing, and presentation. Groups with the presence of SCD were separated using a hierarchal clustering heat map of some hub proteins through hierarchal clustering heat map analysis, separately (Figure 3(d)).

### 3.4. Protein Validation

**Figure 4:**
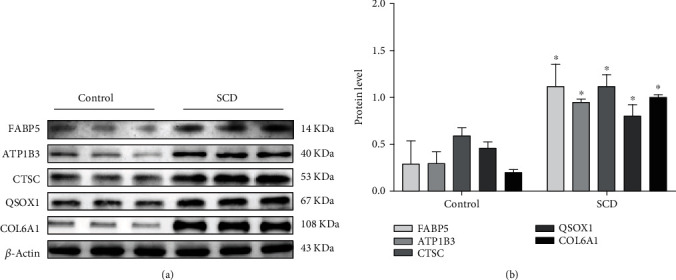
Verification of the expression of genes: (a) Western blotting expression of ATP1B3, COL6A1, CTSC, FABP5, and QSOX1 in the control (Con) and SCD groups. (c) Relative expression of ATP1B3, COL6A1, CTSC, FABP5, and QSOX1 by Western blotting analysis. ^∗^*p* < 0.05 compared with the control group (Student's *t*-test). *β*-Actin was used as a reference protein.

Western blot was conducted to further measure the selected hub proteins (ATP1B3, COL6A1, CTSC, FABP5, and QSOX1). The results of this study showed a significant increase in the SCD group compared to the control (*p* < 0.05). This indicated a consistent protein expression trend with the proteomic results (Figures 4(a) and 4(b)).

### 3.5. The Expression of CTSC and TNF-*α* in CHD and SCD

**Figure 5:**
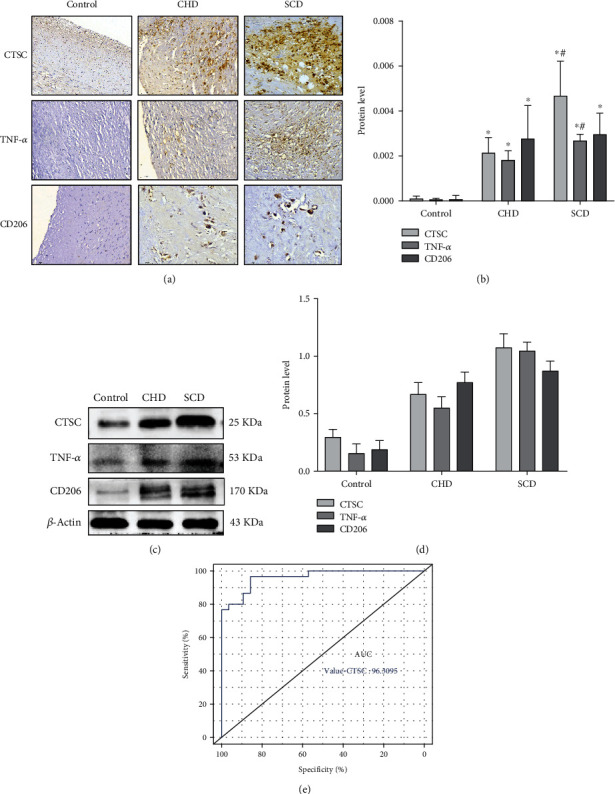
(a, b) Protein expression and cellular localization of CTSC, TNF-*α*, and CD206 in different outcomes of coronary atherosclerotic heart disease. The positive expression in the CHD group and the SCD group was significantly higher than that in the control group. (c, d) The protein expression levels of CTSC, TNF-*α*, and CD206 were determined by Western blot analysis. (e) AUC: area under the ROC curve; ROC: receiver operating characteristic. ^∗^*p* < 0.05 compared with the control group; ^#^*p* < 0.05 compared with the CHD group (Student's *t*-test). *β*-Actin was used as a reference protein.

For the occurrence and development of SCD, IHC was run to localize and determine the expression of CTSC, TNF-*α*, and CD206 in arterial tissues. This was to clarify the possible mechanism behind the involvement of differentially expressed proteins. It was revealed that the positive expression of CTSC was mainly localized in the cytoplasm of foam cells, higher in the atherosclerosis and SCD groups compared with the control (*p* < 0.05; Figures 5(a) and 5(b)). Besides, SCD cases had even higher positive expression of CTSC compared with atherosclerosis cases (*p* < 0.05; Figures 5(a) and 5(b)).

Tumor necrosis factor (TNF-*α*), a marker protein of macrophage M1, was detected in the cytoplasm of foam cells. A significant increase in TNF-*α* was found in the atherosclerosis and SCD groups, compared with the control group (*p* < 0.05) whereas there was no significant difference between the atherosclerosis and SCD cases (*p* > 0.05). This implies that TNF-*α* significantly increased with the increase of CTSC. Further, this indicates an elevated macrophage M1 polarization level (*p* < 0.05), which in turn induces inflammatory reaction.

To study the role of M2 macrophages in coronary heart disease, cell localization and expression of marker protein CD206 in the macrophages were detected by immunohistochemical staining. It was found that CD206 was mainly expressed in the foam cell cytoplasm, positively expressed in tan or brown, compared with the control group. The expression of CD206 in coronary heart disease and sudden death from coronary heart disease groups showed significantly expressed coronary blood vessels in an increasing trend (*p* < 0.05). However, the expression of CD206 in the sudden death of the coronary heart disease group had no significant difference (*p* > 0.05) compared with the coronary heart disease group. The results of WB were the same as those of immunohistochemistry (Figures 5(c) and 5(d)).

Similarly, ROC was plotted, and AUC was recorded, and the results showed AUC of 0.96, suggesting that the CTSC had a good predictive value for SCD, revealing that CTSC had a good differential diagnosis value for CHD and SCD (Figure 5(e)).

### 3.6. The Effects of CTSC on Macrophage Polarization of M1 in SCD via p38/MAPK Signaling Pathway

**Figure 6:**
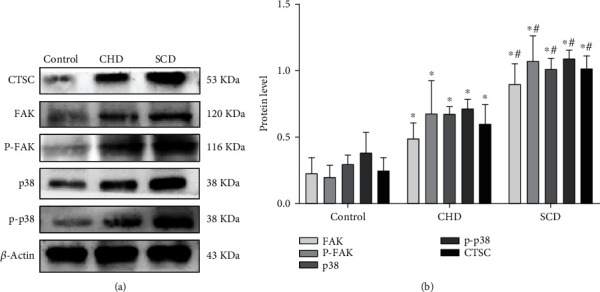
(a, b) Detection results of FAK, p38, and their phosphorylation by Western blot analysis. ^∗^*p* < 0.05 compared with the control group; ^#^*p* < 0.05 compared with the CHD group (Student's *t*-test). *β*-Actin was used as a reference protein.

The expressions of CTSC, FAK, p-FAK, and proteins related to the p38/MAPK signaling pathway were measured by Western blot. This was to explore the mechanism behind the involvement of CTSC in the development of atherosclerosis by regulating macrophage polarization and promoting inflammatory response. It was found that p-FAK and p-p38 were significantly higher in the atherosclerosis and SCD groups compared with the control group and the expressions of FAK, p38, and their phosphorylated forms (*p* < 0.05). This showed a consistent expression trend in the CTSC (Figures 6(a) and 6(b)).

### 3.7. Correlation Analysis

**Figure 7:**
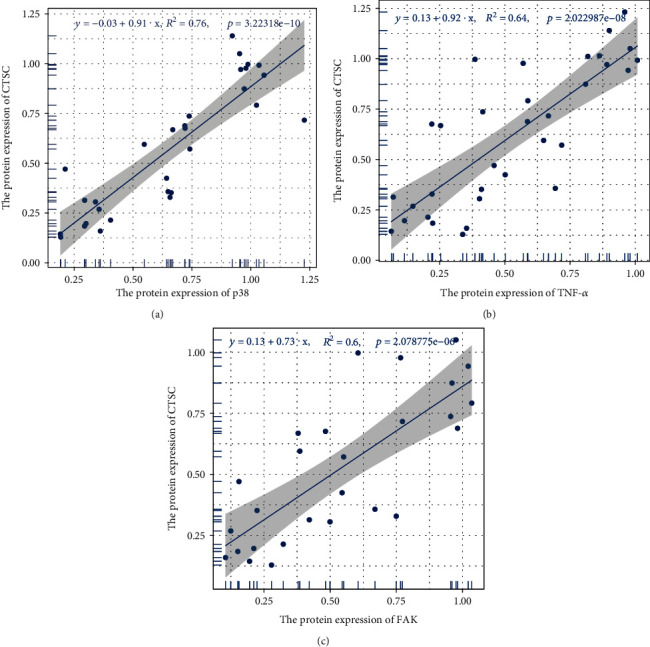
Scatter plots of the linear regression analyses. Scatter plots of the linear regression analyses between CTSC and TNF*α*, CTSC and FAK, and CTSC and p38. *R*^2^ is the determination coefficient.

The Pearson correlation coefficient test method was used to analyze the statistical data of protein expression among CTSC, TNF-*α*, FAK, and p38. This was to display the protein expression relationship among CTSC, TNF-*α*, FAK, and p38 in a more intuitive manner. The results of this study showed that the expression of FAK increased with the increase in CTSC expression, and there was a significant positive correlation between the expression of FAK and CTSC (*R*^2^ = 0.60, *p* = 2.079*e* − 6) (Figure 7(c)). Expression of p38 was positively correlated with CTSC (*R*^2^ = 0.76, *p* = 3.223*e* − 10) (Figure 7(a)) whereas the expression of TNF-*α* was positively correlated with CTSC (*R*^2^ = 0.64, *p* = 2.023*e* − 8). Therefore, CTSC may be activated by FAK to regulate macrophage M1 polarization through the p38/MAPK signaling pathway.

## 4. Discussion

Sudden cardiac death (SCD) is a common type of sudden death in forensic autopsy. However, the pathogenesis of SCD remains elusive. Proteins are characterized by tissue-specific expression patterns [5], and proteomics can make up the defect on impediment of recognition and identification for potential membrane-embedded drug targets dependent solely on RNA [7]. This necessitates more targeted research into biomarkers. In this study, a proteomic profile of the human left anterior descending coronary artery was explored to identify proteins potentially involved in major pathophysiological processes related to SCD.

A corresponding network model was constructed by label-free quantitative proteomics. From the constructed network model, a total of 265 differentially expressed proteins were found in the SCD and control individuals. In the hub modules, a significant increase in the expressions of cathepsin family members (CTSZ, CTSC, CTSF, CTSD, CTSB, and CTSA) which are related to inflammation and lysosomal pathways was observed in the SCD group. Consequently, this indicated that the cathepsin family can be a potential biomarker of SCD.

Cathepsins are a large class of hydrolytic proteases widely expressed in lysosome. Currently, more than 20 kinds of cathepsins have been discovered, including 11 kinds of human cathepsins [8]. They play an indispensable role in autophagy, antigen presentation, cell stress signal transfer, metabolism, and lysosome-dependent cell death. Studies have shown that multiple cathepsins are involved in the occurrence and development of atherosclerosis [9, 10]. Macrophages and derived foam cells are the main carriers of cathepsins which are almost seen in the subintima of arteries. It has been reported that most cathepsins are expressed as atherosclerosis progresses and correlate with plaque instability and fiber cap rupture.

Cathepsin C (CTSC) is also known as dipeptidyl peptidase I (DPPI), a cysteine-type cathepsin that is widely expressed in immune cells, such as myeloid cells, cytotoxic T lymphocytes, mast cells, neutrophils, macrophages, and their precursors. It plays biological functions in cascade reactions as a result of interactions with other signal molecules, which are mainly reflected in promoting inflammatory reaction by activation of proinflammatory granzyme, a kind of serine protease [11]. In this study, we found that the expression of CTSC was mainly localized in the cytoplasm of foam cells and upregulated in coronary heart disease, and it also had a good differential diagnosis value for CHD and SCD via ROC. This is in consistence with the finding by Herías' team [12] in 2015 that shows an upward trend in the expression of CTSC in human carotid atherosclerotic plaque. To clarify the role of CTSC in the occurrence of SCD, this study set up a SCD group. In this group, the expression of CTSC was significantly higher compared with expression in other groups. Further, this study also identified the active action of CTSC on SCD occurrence and development.

Atherosclerosis is a chronic inflammatory arterial disease that involves a variety of inflammatory factors [13–15]. Hyperlipidemia and inflammation are regarded as the two main predisposing factors of atherosclerosis [16]. Inflammatory factors are important players in the occurrence and development of diseases that are mainly released by infiltrating macrophages. Macrophages are the main medium in driving the occurrence and development of atherosclerosis and basically run through the whole process of disease development. They can present with diverse phenotypes or polarization states depending on their types and functions (type M1 and type M2).

Macrophage M1 functions to potentiate inflammatory reactions by expressing inflammatory cytokines, whereas macrophage M2 devotes to degrading or inhibiting inflammatory factors to reversely inhibit the occurrence and aggravation of inflammatory reactions [17–19]. A previous cell detection observed that CTSC can advance the process of inflammatory reaction because it can increase the release of inflammatory factors by regulating macrophage polarization to M1 [20]. Further, it was found that inflammatory reaction is the pathological basis of the development of atherosclerosis. In our previous studies, we demonstrated a significant increase of CTSC and macrophage M1 marker TNF-*α* in atherosclerosis cases compared with the control group [21]. It was explained that the synergistic effect of CTSC and TNF-*α* may promote the inflammatory response in atherosclerotic lesions and be related to the decreased stability of atherosclerotic lesions. To evaluate the mechanism by which CTSC involves in atherosclerosis development to cause SCD via regulating macrophage polarization and promoting inflammatory response, Western blot and IHC were conducted to measure the expressions of CTSC, TNF-*α*, CD206, FAK, p-FAK, and proteins associated with the p38/MAPK signaling pathway.

Mitogen-activated protein kinase (MAPK) is a well-known class of serine/threonine protein kinases mainly expressed in mammalian cells. Its main constituents are extracellular signal-regulated kinases (ERK), c-Jun N-terminal kinases (JNK), and p38. Activation of p38/MAPK has been identified in patient tissues and animal models through immunohistochemical analysis of macrophages associated with atherosclerotic plaque [22]. In addition, p38/MAPK in most *in vitro* studies has been proven to be a part of the positive feedback mechanism that drives foam cell formation in atherosclerosis. It does this mainly by promoting macrophage proliferation which advances inflammatory responses and facilitates the progress of chronic inflammatory diseases [23].

Focal adhesion kinase (FAK) is a cytoplasmic nonreceptor tyrosine kinase that works on cellular signal transmission through phosphorylation of downstream substrates. It is associated with the transcription of multiple proinflammatory factors as a result of being an upstream mediator. In the nervous system, CTSC helps for microglia M1 polarization via the PKC/p38 (MAPK)/NF-*κ*B axis, resulting in increased release of inflammatory factors, such as CD16, CD32, TNF-*α*, and IL-1*β*, and finally leading to the development of neuroinflammation [24]. In hepatocellular carcinoma, CTSC increases proliferation of cancer cells and migration and decreases apoptosis. This is because CTSC activates the TNF-*α*/p38 (MAPK) pathway and enhances the expression of downstream factors, such as MMP3, MMP9, TNF-*α*, and vimentin [25].

Alam et al. [20] carried out cytological research to evaluate the upregulation of CTSC expression regulating the macrophage polarization to M1 via the FAK-mediated p38/MAPK/NF-*κ*B pathway. According to Alam et al., upregulation of CTSC facilitates the inflammatory factors to release and in turn promote inflammatory response. In our study, the expressions of FAK, p38, and their phosphorylated form p-FAK were found. Further, it was found that p-p38 (downstream effector molecule) was significantly increased with an increase in CTSC in both atherosclerosis and SCD groups, but a higher expression was found in the SCD group. By detecting the expression of M1 macrophage marker protein TNF-*α*, it was found that when the expression of CTSC was increased, the p38/MAPK signaling pathway was activated. This then increased the expression of TNF-*α*, but the expression of CD206 was not significantly affected. We hypothesized that CTSC activation by FAK favors macrophage polarization to M1 through the p38/MAPK signaling pathway. This increases the release of inflammatory factors and thus aggravates inflammation and atherosclerosis.

This study had some limitations including that the sample size of this was relatively small and may have limited identification of the proteins associated with SCD. For example, CTSC affects the polarization of macrophage M1 through the p38/MAPK pathway. Currently, we have only done human samples. For further study, we need to add animal and cytological experiments and explore the specific mechanism of this protein in the pathway by knockout or overexpression of CTSC in the experimental model. In addition, CTSC contains a signal peptide which can be secreted by a variety of cells, but the role of secreted CTSC in the development of atherosclerosis is still a mystery that requires further study.

In conclusion, atherosclerosis is significant in the occurrence and development of SCD. This study has established a proteomic network dependent on proteomic changes and identified potential biomarkers involved in SCD pathogenesis. It shows that CTSC regulates macrophage M1 polarization through the p38/MAPK signaling pathway to participate in the inflammatory response. Therefore, the findings of this study provide complementary information on the current conclusions at the cytological level.

## Data Availability

The datasets used and analyzed during the present study are available from the corresponding author on reasonable request.

## Supplementary Materials

Supplementary Materials As shown in Supplementary Figure 1, the quantitative principal component analysis results of 6 samples show that the higher the degree of aggregation between repeated samples, the better the quantitative repeatability.
