# Supplementary material for: Cathepsin C Is Involved in Macrophage M1 Polarization via p38/MAPK Pathway in Sudden Cardiac Death
Source: Cardiovasc Ther. 2021 Oct 15;2021:6139732. doi: 10.1155/2021/6139732 (PMC8536465; doi:10.1155/2021/6139732)
Supplement: Supplementary Materials — As shown in Supplementary Figure 1, the quantitative principal component analysis results of 6 samples show that the higher the degree of aggregation between repeated samples, the better the quantitative repeatability. [file 6139732.f1.doc]

Supplementary Figure legends


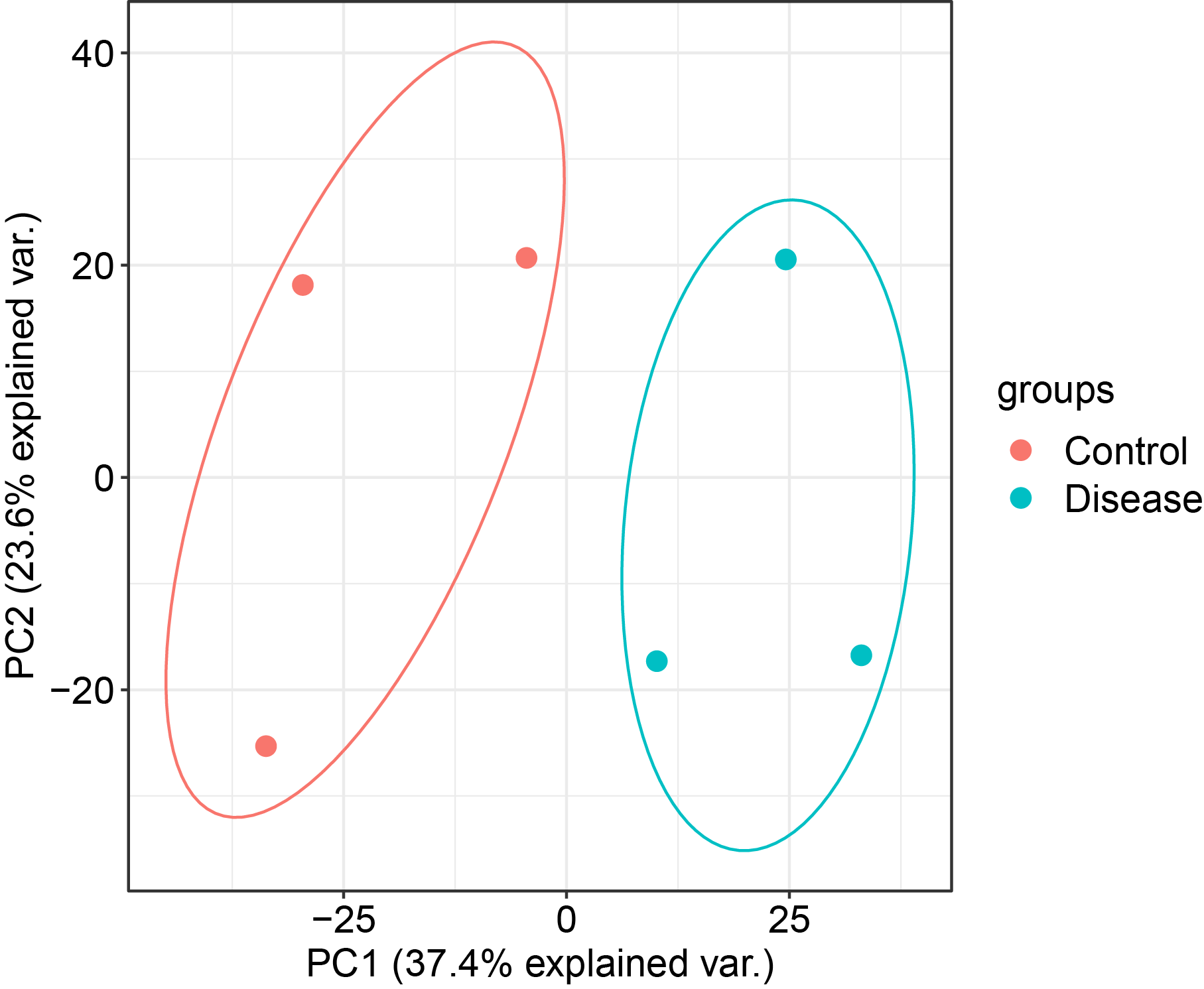


Figure 1: The quantitative principal component analysis results of 6 samples were shown in the figure. The higher the degree of aggregation between repeated samples, the better the quantitative repeatability (Source: *Dai, J., et al., Significances of viable synergistic autophagy-associated cathepsin B and cathepsin D (CTSB/CTSD) as potential biomarkers for sudden cardiac death. BMC Cardiovasc Disord, 2021. 21(1): p. 233.*).
